# Artificial intelligence significantly facilitates development in the mental health of college students: a bibliometric analysis

**DOI:** 10.3389/fpsyg.2024.1375294

**Published:** 2024-03-07

**Authors:** Jing Chen, Dongfeng Yuan, Ruotong Dong, Jingyi Cai, Zhongzhu Ai, Shanshan Zhou

**Affiliations:** ^1^Wuhan University China Institute of Boundary and Ocean Studies, Wuhan, China; ^2^Faculty of Pharmacy, Hubei University of Chinese Medicine, Wuhan, China; ^3^Hubei Shizhen Laboratory, Wuhan, China; ^4^The First Clinical Medical School, Hubei University of Chinese Medicine, Wuhan, China

**Keywords:** mental health, college students, artificial intelligence, machine learning, bibliometric

## Abstract

**Objective:**

College students are currently grappling with severe mental health challenges, and research on artificial intelligence (AI) related to college students mental health, as a crucial catalyst for promoting psychological well-being, is rapidly advancing. Employing bibliometric methods, this study aim to analyze and discuss the research on AI in college student mental health.

**Methods:**

Publications pertaining to AI and college student mental health were retrieved from the Web of Science core database. The distribution of publications were analyzed to gage the predominant productivity. Data on countries, authors, journal, and keywords were analyzed using VOSViewer, exploring collaboration patterns, disciplinary composition, research hotspots and trends.

**Results:**

Spanning 2003 to 2023, the study encompassed 1722 publications, revealing notable insights: (1) a gradual rise in annual publications, reaching its zenith in 2022; (2) *Journal of Affective Disorders* and *Psychiatry Research* emerged were the most productive and influential sources in this field, with significant contributions from China, the United States, and their affiliated higher education institutions; (3) the primary mental health issues were depression and anxiety, with machine learning and AI having the widest range of applications; (4) an imperative for enhanced international and interdisciplinary collaboration; (5) research hotspots exploring factors influencing college student mental health and AI applications.

**Conclusion:**

This study provides a succinct yet comprehensive overview of this field, facilitating a nuanced understanding of prospective applications of AI in college student mental health. Professionals can leverage this research to discern the advantages, risks, and potential impacts of AI in this critical field.

## 1 Introduction

College students are frequently selected as subjects for psychological research, primarily because researchers from universities or research institutes are more easily accessed to them. Moreover, college students usually have relatively mature cognitive abilities and a willingness to cooperate, which can quickly participate in and conduct experiments, thereby promoting the development of scientific research work (Chen and Jiang, 2019; Wang et al., 2020). In recent years, surveys have shown a continuous increase in the prevalence of depression and anxiety symptoms among college students (Liu et al., 2019; Lipson et al., 2019). Globally, approximately 31% of college students show positive results in mental health disorder screenings (Auerbach et al., 2018), attracting widespread social attention. The factors contributing to mental health disorders in college students are diverse, and the pathological development of common mental disorders such as depression and anxiety is complex. It is challenging to determine their origins, diagnosis, prognosis, and treatment processes (Alexopoulos, 2005; Greenberg et al., 2012; Slimmen et al., 2022). Despite the rapid development of clinical psychology and psychiatry in recent decades, diagnostic ambiguity and uncertain prognosis persist, with psychological and pharmacological treatments typically effective in only 30 to 50% of patients (Stein et al., 2022; Hoeflich et al., 2023). The majority of patients remain in a state where timely detection and treatment are challenging (Colizzi et al., 2020). Therefore, the field of college student mental health urgently needs comprehensive diagnosis and prediction based on multidimensional data to provide targeted interventions and treatments (Torous and Keshavan, 2018), all of which require effective analysis based on big data.

The rapid advancement of technologies such as neuroimaging, social media, smartphones, and wearable devices has enabled practitioners in the mental health field to quickly access vast amounts of data (Benoit et al., 2020). Artificial intelligence (AI), as a computational technology mimicking human intelligence, utilizes advanced algorithms to automatically learn from this data, making it easier and more accurate to identify individualized data patterns and make precise predictions from massive multidimensional data (Ramesh et al., 2004). Currently, AI technologies have played a significant role in various fields such as robotics, image and speech recognition, natural language processing, and expert systems (Sheikhtaheri et al., 2014; Trivikram et al., 2017; Yu et al., 2018; Xiang et al., 2020). Additionally, machine learning has demonstrated promising applications in the diagnosis, treatment, and prognosis prediction of mental health disorders (Gao et al., 2018; Abd-Alrazaq et al., 2023). Moreover, AI can be directly or indirectly applied to intelligent digital therapies (Minerva and Giubilini, 2023). For example, AI is used in conjunction with electronic health records, brain imaging, sensor based monitoring systems, and social media platforms to predict, classify, or subgroup mental illnesses and suicide, providing interventions for preventing and treating mental health disorders (Lee et al., 2021). These studies indicate that leveraging AI technologies for processing mental health big data holds profound significance for the development and progress of the mental health field (Noorbakhsh-Sabet et al., 2019).

As AI rapidly transforms the landscape of medicine, academic research on the intersection of AI and college student mental health has significantly increased in recent years (Gil et al., 2022; Wang, 2023). This necessitates a comprehensive review of the research trends in this field. Bibliometric methods demonstrate unique advantages in evaluating global scientific production and developmental trends. They offer an opportunity to understand information within a research field and facilitate interdisciplinary collaboration. As a measurable information science method, bibliometrics analyze emerging trends and knowledge structures within a field to obtain quantifiable, repeatable, and objective data (Ai et al., 2023; Kuang et al., 2023). Although some reviews have summarized the application of AI in the mental health of college students (Abd-Alrazaq et al., 2024), bibliometric methods have not yet been applied to this research field. Qualitative reviews may carry potential subjective bias (Hosseini et al., 2018), so it is necessary to emphasize the use of quantitative methods to conduct a systematic review of research on AI and college students’ mental health. This study aims to provide an overview of research in the AI and college student mental health field over the past two decades using bibliometric methods. By better understanding publication and research trends, it summarizes current research hotspots, status, and future challenges in the field, proposing potential directions to address these challenges.

## 2 Methods

### 2.1 Data sources

During this study, the four major databases of Web of Science (WoS), PubMed, Scopus, and Google Scholar were considered. The search found that WoS contains the most literature, and after screening and removing duplicates, the main source of literature is still WoS. WoS has higher quality literature and significantly better citation accuracy than other databases (Falagas et al., 2008; Wang and Waltman, 2016; Yuan et al., 2024). Therefore, this article chooses to extract data from the WoS database.

We obtained pertinent studies pertaining to AI and the mental health of college students from the WoS core dataset. In order to construct a comprehensive database for research on AI and mental health among college students, our retrieval process was centered on three key aspects. Firstly, to broadly obtain studies related to mental health, the WOS search expression was set as follows: TS = (“mental health” OR “mental disorder*” OR “mental wellness” OR “mental illness*” OR “depression” OR “stress” OR “anxiety” OR “bipolar disorder” OR “borderline personality disorder” OR “schizophrenia”). Secondly, to restrict the research object to college students, the WOS search expression was set as follows: TS = (“university student*” OR “college student*” OR “higher education” OR “undergrad student*” OR “master’s student*” OR “postsecondary education” OR “undergraduate*” OR “tertiary education” OR “postsecondary education” OR “doctoral student*” OR “Ph.D. student*”). Thirdly, to highlight the application of AI in college student mental health, the WOS search expression was set as follows: TS = (“machine learning” OR “artificial intelligence” OR “deep learn*” OR “neural network” OR “convolutional neural network” OR “recurrent neural networks” OR “natural language process*” OR “CNN” OR “LSTM” OR “RNN” OR “AI” OR “support vector machine” OR “logistic regression” OR “random forest” OR “naïve bayes” OR “decision tree”). The Boolean operator *OR* was used between keywords in all three searches, and Boolean operator *AND* was used between three topics. In addition, asterisks were used as wildcards to explain various variations of several keywords.

### 2.2 Data screening

**Figure 1:**
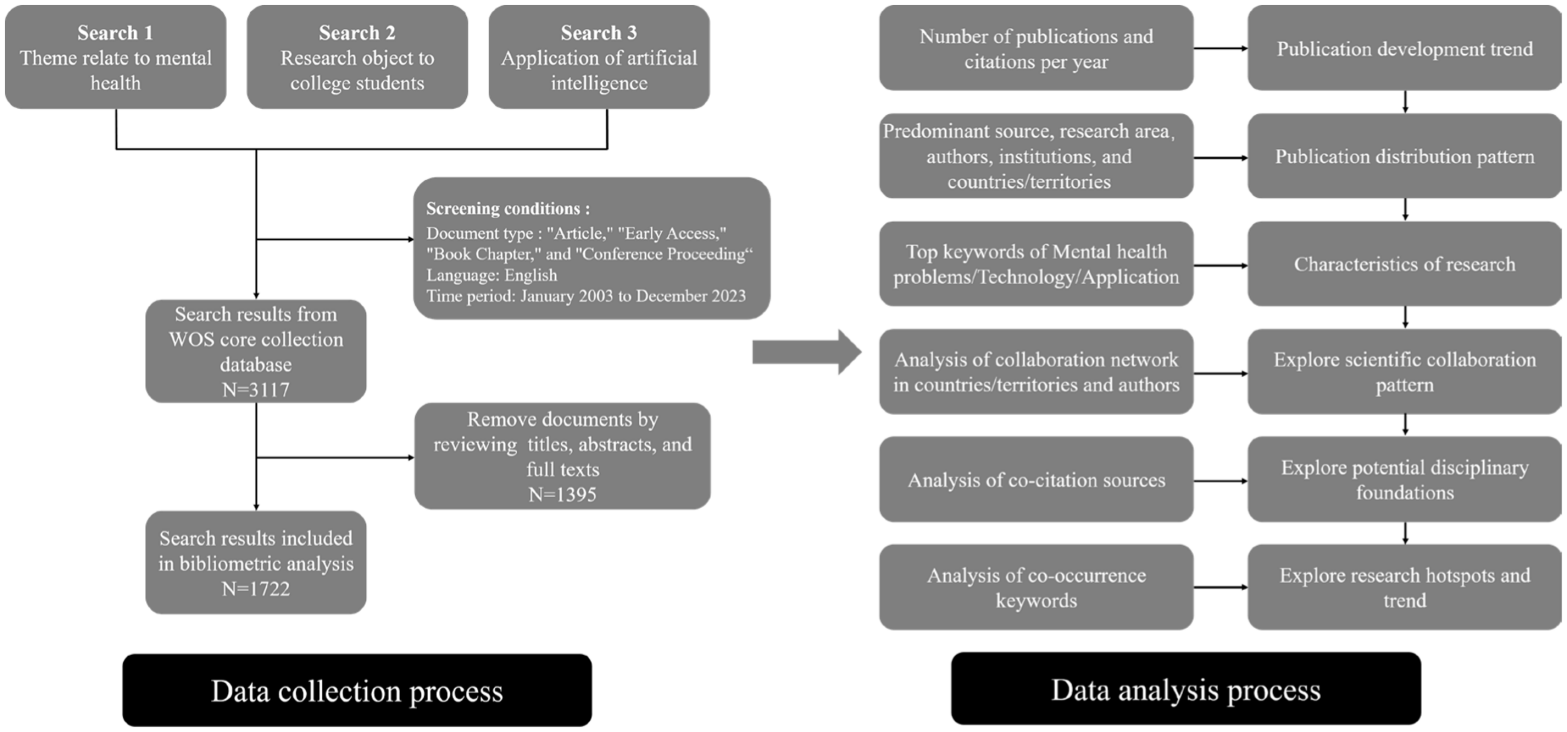
Data collection and screening process.

The screening methodologies were implemented as follows: Document type restrictions were applied to include “Article,” “Early Access,” “Book Chapter,” and “Conference Proceedings.” The time span was delimited from January 2003 to December 2023, and the language criterion was set to English. As of December 29, 2023, a total of 3,117 articles were retrieved. Furthermore, throughout the entire process of data screening and collection, two independent reviewers (J. Chen and D. Yuan) identified potential studies based on inclusion and exclusion criteria by scrutinizing article titles, abstracts, and full texts. Any disparities that emerged were deliberated upon and resolved through discussion, resulting in a consensus. All data for this study were extracted by December 31, 2023, and following the screening, a total of 1,722 articles were deemed eligible for inclusion. The data selection analysis process is delineated in Figure 1.

### 2.3 Data analysis

Bibliometrics, as a quantitative analysis of literature, serves as a methodological tool to delineate developmental trends within specific academic fields, emphasizing the attainment of quantifiable, replicable, and objective results. In this study, we conducted an analysis of annual publication counts and citation frequencies to elucidate the evolutionary patterns within the field over the past two decades. The growth rate, a pivotal metric portraying developmental trends (Guo et al., 2020), was calculated based on the increase in publications over time relative to the baseline year of 2003. It is noteworthy that while the growth rate signifies the rate of change in publications relative to 2003, it does not provide insight into the proportion of AI-related publications among all publications. Additionally, utilizing data from the (WoS), we examined citation data for the top 10 journals, research domains, countries, institutions, and authors with respect to publication quantity. This investigation aimed to provide insights into the distribution and predominant productivity of publications in the domain of AI and college student mental health.

Growth rate (%) = [(number of publications in the last year ÷ number of publications in the first year)^1/(last year - first year)^ - 1] × 100%.

We employed VOSviewer software, a widely recognized tool for bibliometric analysis, to visually examine literature. VOSviewer utilizes the interrelationships among academic entities such as authors, journals, and keywords to depict scientific research trends within a specific domain. Analysis units in VOSviewer encompass countries, authors, journals, keywords, and more, tailored to the analytical focus and database type. Nodes in the visualization represent analysis units, with node size indicating their significance in the network. Connections between nodes are depicted by lines, with thicker lines denoting stronger connections. Node colors denote distinct clusters, where nodes of the same color belong to the same cluster (Hernandez-Torrano et al., 2020). In this study, we initially analyzed collaborative networks in countries/territories and authors to elucidate patterns of scientific collaboration in AI and college student mental health. Subsequently, a co-citation analysis based on publication sources was conducted to reveal potential disciplinary compositions within this field. Finally, through visual examination of keywords, we explored research hotspots and developmental trends in the domain.

## 3 Results

### 3.1 Developmental trends in AI research on college students mental health

**Figure 2:**
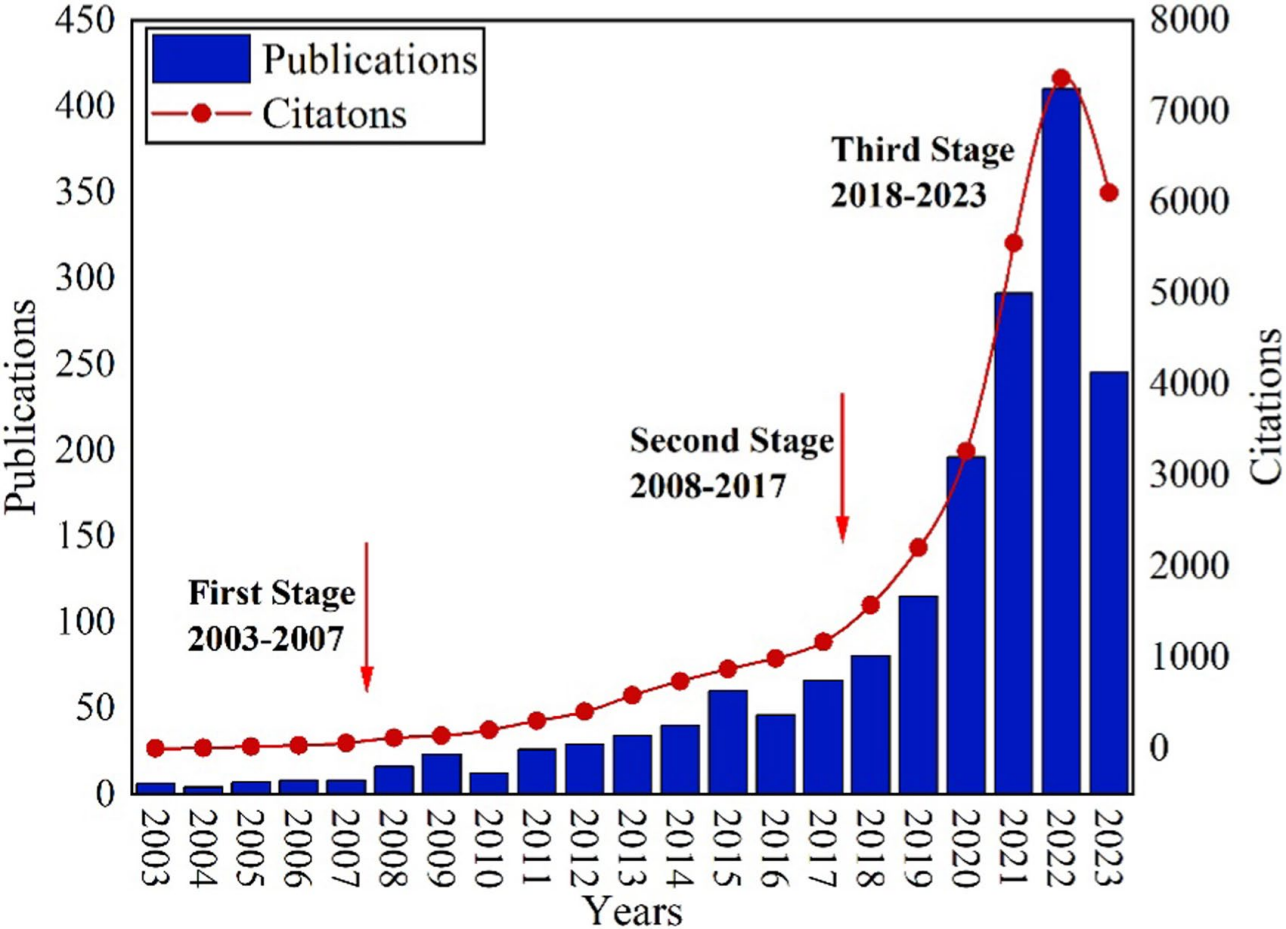
The distribution of the publications and citations per year.

Figure 2 illustrates the annual evolution of publications and citation frequency associated with AI and college student mental health research from 2003 to 2023. The research trajectory in this field can be roughly divided into three stages: First Stage (2003–2007): During this initial period, the volume of studies focusing on AI and college student mental health was relatively modest. The growth rate from 2003 to 2007 stood at 7.46%, indicating a comparatively subdued level of attention and interest from researchers in this field. Second Stage (2008–2017): Noteworthy increases were observed in both publications and citation frequency during this phase, with a growth rate of 17.05%. This suggests that, with the development of AI, the application of AI in college student mental health gradually gained attention. Third Stage (2018–2023): Both the publications and citation frequency showed significant increases and publications during this timeframe constituted 75.45% of the total papers (1,337/1772), with a growth rate of 50.46% from 2018 to 2022. In 2022, the pinnacle was reached with the highest number of publications (410) and citation frequency (7362). While there was a slight downturn in both the annual publications and citations in 2023, they remained at an elevated level.

### 3.2 Publication distribution for AI in mental health of college students

#### 3.2.1 Predominant publication source

Table 1 presents the foremost 10 core publication sources in terms of publication quantity within the field. Collectively, these journals published 452 papers, constituting 25.51% of the total literature. The J*ournal of Affective Disorders* emerged as the most influential contributor, presenting 77 studies in the field of AI and college student mental health over the past two decades, encompassing 4.47% of the total literature and ranking first. *Frontiers in Psychology*, a specialized journal in psychology, secured the second position by publishing 89 articles, while the *International Journal of Environmental Research and Public Health* secured the third position with 53 publications. Notably, despite its interdisciplinary and relatively recent establishment, *Plos One* ranked the fourth position by contributing significantly to studies in this field. Moreover, journals pertaining to psychiatry and public health played pivotal roles in the field’s advancement. Two specialized psychiatry journals, *BMC Psychiatry* and *Frontiers in Psychiatry,* shared the sixth position by each published 38 pertinent studies. Two specialized public health journals, B*MC Public Health* (35, 2.03%) and *Frontiers in Public Health* (33, 1.92%), secured the eighth and ninth positions, respectively. In 2022, the Impact Factors (IF) of the aforementioned top 10 journals ranged from 2 to 12, with 40% JCR (Journal Citation Reports) partition falling within Q1, signifying the reliability and high quality of these studies. Intriguingly, *Psychiatry Research*, despite ranking tenth with only 21 published studies, exhibited the highest average citation frequency of 37.1, indicating its potential status as the most widely recognized and esteemed journal in the field.

**Table 1:** The distribution of publications by top 10 publication sources.

Publication sources	Publications (%)	Citations	Avergae citations	H-index	IF (2022)	JCR (2022)
Journal of Affective Disorders	77 (4.47)	2007	26.06	22	6.6	Q1
Frontiers in Psychology	64 (3.72)	780	12.19	13	3.8	Q1
International Journal of Environmental Research And Public Health	53 (3.08)	785	14.81	16	-	-
PLoS One	52 (3.02)	1882	36.19	20	3.7	Q2
Journal of American College Health	41 (2.38)	676	16.49	13	2.4	Q2
BMC Psychiatry	38 (2.21)	1,008	26.53	13	4.4	Q2
Frontiers In Psychiatry	38 (2.21)	379	9.97	12	4.7	Q2
BMC Public Health	35 (2.03)	1,275	36.43	18	4.5	Q2
Frontiers in Public Health	33 (1.92)	131	3.97	7	5.2	Q1
Psychiatry Research	21 (1.22)	779	37.1	11	11.3	Q1

#### 3.2.2 Predominant research areas

Table 2 presents the major contributing fields to AI and college student mental health research. The analysis reveals that nearly half of the publications are found in journals related to Psychiatry (443, 25.73%) and Public Environmental Occupational Health (346, 20.09%). Journals in the field of Psychology also exhibit substantial influence, publishing 322 studies, constituting almost 18.67% of the publications. Similarly, journals in the fields of Neurosciences Neurology and Computer Science have accumulated over 120 relevant studies. Other related research in this field is primarily concentrated in the domains of education, medicine, and engineering technology, including Education Educational Research, Science Technology Other Topics, General Internal Medicine, Engineering, and Health Care Sciences Services.

**Table 2:** The distribution of publications by top 10 research areas.

Research areas	Publications (%)	Citations	Avergae citations	H-index
Psychiatry	443 (25.73)	10,637	24.01	49
Public Environmental Occupational Health	346 (20.09)	6,212	17.95	40
Psychology	322 (18.67)	6,392	19.85	40
Neurosciences Neurology	159 (9.23)	3,806	23.94	32
Computer Science	123 (7.14)	869	7.07	16
Education Educational Research	114 (6.62)	1967	17.25	21
Science Technology Other Topics	103 (5.98)	2,447	23.76	23
General Internal Medicine	101 (5.87)	1786	17.68	21
Engineering	81 (4.70)	794	9.8	15
Health Care Sciences Services	79 (4.59)	1,357	17.18	19

#### 3.2.3 Predominant authors, institutions, and countries/territories

Globally, a total of 107 countries/territories have contributed to research in the field of AI and college student mental health. Table 3 delineates the top 10 countries in terms of publication quantity. The leading five countries or territories in terms of paper quantity include China, the United States, Australia, England, and Canada. China (483, 28.05%) and the United States (459, 26.66%) together account for over the half hail from the publications, ranking the first and second, respectively, in an absolute leading position in this field. Australia follows with 81 published studies (4.70%), and England with 78 articles (4.53%). Canada contributed 73 studies (4.24%). Other countries listed in the table are primarily located in Asia (Japan, India, South Korea, Thailand). Overall, research related to AI and college student mental health has garnered varying levels of attention worldwide, particularly in developed countries. When considering the average citation frequency for each paper, the USA (26.39) leads, followed by Australia (23.52) and Spain (22.42). Notably, despite having the highest number of publications, China’s average citation frequency (13.31) is notably lower than that of other countries. Therefore, there is a need to increase research investment and produce more high-quality research to enhance China’s international influence and competitiveness in this field.

**Table 3:** The distribution of publications by top 10 countries/territories, institutions, and authors.

	Rank	Name	Publications	Citations	Average citation	H-index
Country/Territories	1	China	483 (28.05)	6,429	13.31	39
	2	United States	459 (26.66)	12,114	26.39	54
	3	Australia	81 (4.70)	1905	23.52	24
	4	England	78 (4.53)	1,397	17.91	22
	5	Canada	73 (4.24)	1,496	20.49	19
	6	Spain	69 (4.01)	1,547	22.42	20
	7	Japan	66 (3.83)	1,150	17.42	16
	8	India	62 (3.60)	820	13.23	13
	9	South Korea	57 (3.31)	775	13.6	15
	10	Thailand	56 (3.25)	953	17.02	18
Institution	1	Harvard University	52 (3.02)	2,561	49.25	23
	2	Mahidol University	36 (2.09)	785	21.81	16
	3	Ciber Centro De Investigacion Biomedica En Red	34 (1.97)	791	23.26	13
	4	University of California System	34 (1.97)	1,167	34.32	12
	5	University of Limpopo	34 (1.97)	812	23.88	17
	6	University of Southern California	30 (1.74)	449	14.97	10
	7	University of Michigan	28 (1.63)	1,333	47.61	15
	8	Central South University	26 (1.51)	530	20.38	8
	9	Peking University	26 (1.51)	456	17.54	11
	10	Human Sciences Research Council South Africa	24 (1.39)	664	27.67	13
Researcher	1	Peltzer Karl	36 (2.09)	839	23.31	17
	2	Pengpid Supa	35 (2.03)	793	22.66	16
	3	Oh Hans	16 (0.93)	100	6.25	6
	4	Koyanagi Ai	15 (0.87)	366	24.4	8
	5	Zhang Yan	15 (0.87)	91	6.07	6
	6	Zhou Sasha	15 (0.87)	123	8.2	6
	7	Wang Ying	14 (0.81)	178	12.71	6
	8	Kessler Ronald C	13 (0.76)	400	30.77	7
	9	Tzourio Christophe	13 (0.76)	161	12.38	8
	10	Yang Tingzhong	11 (0.64)	133	12.09	6

Over the past two decades, a total of 2,576 research institutions globally have engaged in research on AI and college student mental health. Table 3 presents the top 10 research institutions in terms of publication quantity. Harvard University from the United States (53, 3.02%) holds the top position, closely followed by Mahidol University in Thailand (36, 2.09%). Remarkably, the third-ranked institution is CIBER (Centro de Investigación Biomédica en Red) from Spain. This institution is committed to establishing a multidisciplinary, cross-team collaborative network to enhance the collaborative research of biomedical research, improving research efficiency and quality. Over the past two decades, it has published a total of 34 studies, making significant contributions to collaborative research in this field. Among the top 10 institutions in terms of publication quantity, over half hail from the United States and China, potentially explaining the leading publication numbers of these countries compared to others. Regarding average citation frequency, the top three research institutions from the United States–Harvard University (49.25), University of Michigan (47.61), and University of California System (34.32)–surpass research institutions from other countries, highlighting the strong international influence of the United States in this field, with its research outcomes widely recognized by international peers.

Globally, over the past two decades, a total of 7,769 researchers have participated in research on AI and college student mental health. Table 3 lists the top 10 researchers in terms of publication quantity. The top 10 authors collectively have published 183 studies, constituting 10.33% of the total research in this field. Prof. Karl Peltzer stands out as the most prolific researcher in this field, publishing 36 related studies and ranking first. Following closely are Prof. Supa Pengpid and Prof. Hans Oh, with 35 and 16 related studies, ranking second and third, respectively. Additionally, Prof. Ronald C. Kessler boasts an average citation frequency of 30.77, surpassing other researchers in this field, indicating his status as the most influential researcher.

### 3.3 Characteristics of research for AI in mental health of college students

Keywords Are The author’s extraction and summarization of core terms In a research study. In this study, VOSviewer software Was used To calculate The frequency of keyword occurrence and Total link strength (TLS). TLS represents The total co-occurrence frequency of a keyword with other keywords; The larger The value, The greater The importance of The node In The network graph (Kuang et al., 2023). Table 4 Presents information about The frequency of keyword occurrence and TLS. In The field of AI and college student mental health, The Top five mental health issues Are depression, anxiety, stress, mental disorders, and major depression. Among them, depression Has The highest TLS of 872, indicating Its crucial role In college student mental health issues. The Top five AI technologies Are machine learning, AI, deep learning, random forest, and decision tree. Machine learning Has The highest TLS of 149, signifying Its prominence As The most important technology In this field. The Top five applications of AI In college student mental health Are risk factors, prediction, assessment, clustering, and digital health. Among them, risk factors have The highest TLS of 142, indicating that using AI technology To discover risk factors for college student mental health Is One of The most important applications In this field.

**Table 4:** The top keywords of artificial intelligence in mental health of college students.

Category	Frequency	Total link strength (TLS)
Mental health problems		
Depression	312	872
Anxiety	193	635
Stress	81	239
Major depressive disorder	13	23
Mental disorders	12	25
Technology		
Machine learning	62	149
Artificial intelligence	32	59
Deep learning	19	18
Random forest	10	23
Decision tree	8	19
Application		
Risk factors	60	142
Prediction	9	33
Assessment	7	8
Clustering	5	11
Digital health	5	25

### 3.4 Analysis of scientific collaboration network for AI in mental health of college students

**Figure 3:**
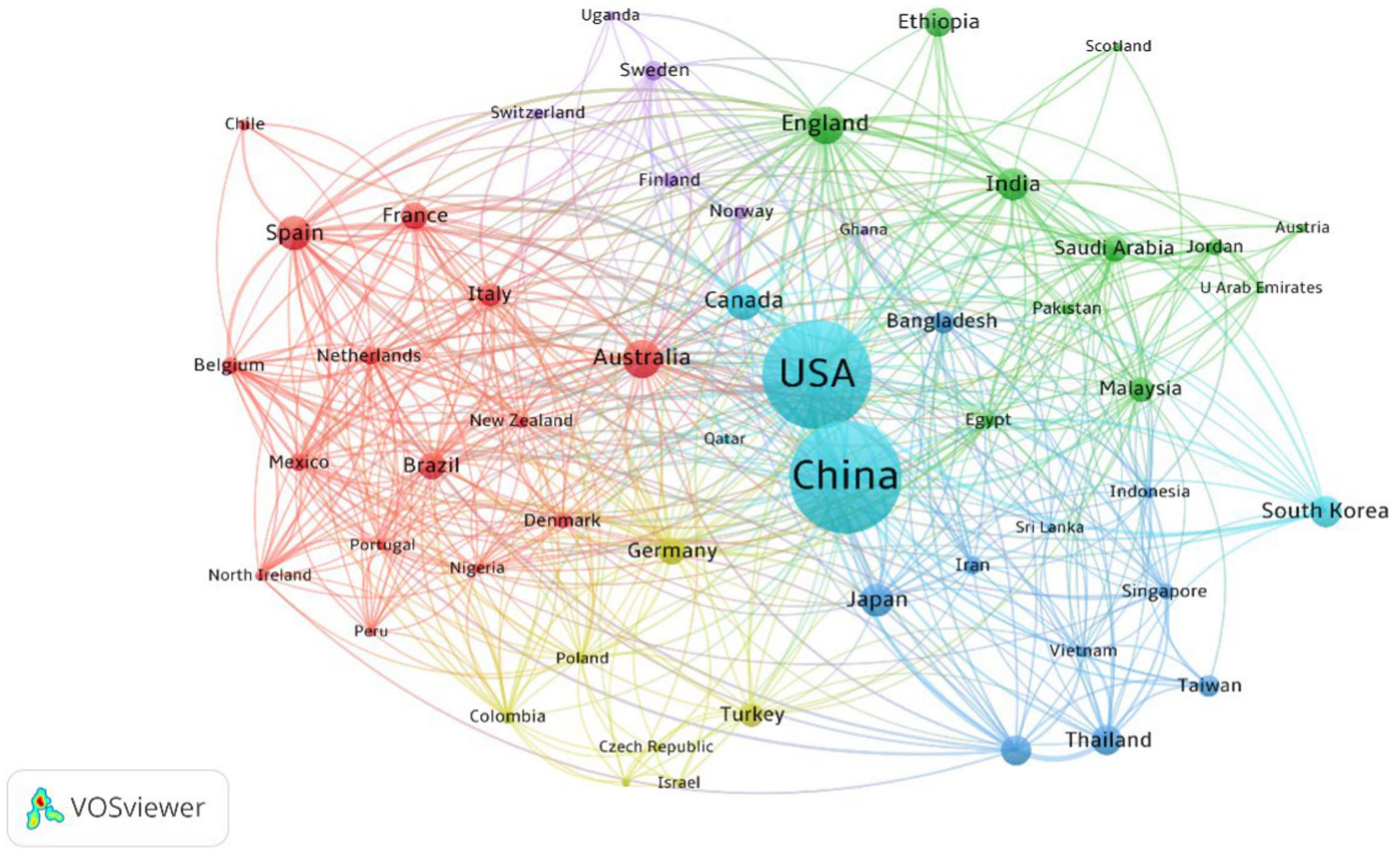
Visualization knowledge maps of the co-authorship between countries/territories.

In this study, we employed VOSviewer software to visualize the global scientific collaboration network in the field of AI and college student mental health from 2003 to 2023, encompassing countries/territories and researchers. Figure 3 delineates the international collaboration network in this field, with lines connecting nodes indicating cooperative relationships among countries/territories. Thicker lines denote closer collaboration. This analysis includes only countries that have contributed a minimum of 6 articles to the dataset, resulting in 107 nodes and 535 connections. China and the United States, positioned as the largest nodes, hold central roles in the network graph, establishing robust collaborative ties with other countries or territories (such as Canada and South Korea) within the Light Blue cluster, underscoring their significant influence in this field.

**Figure 4:**
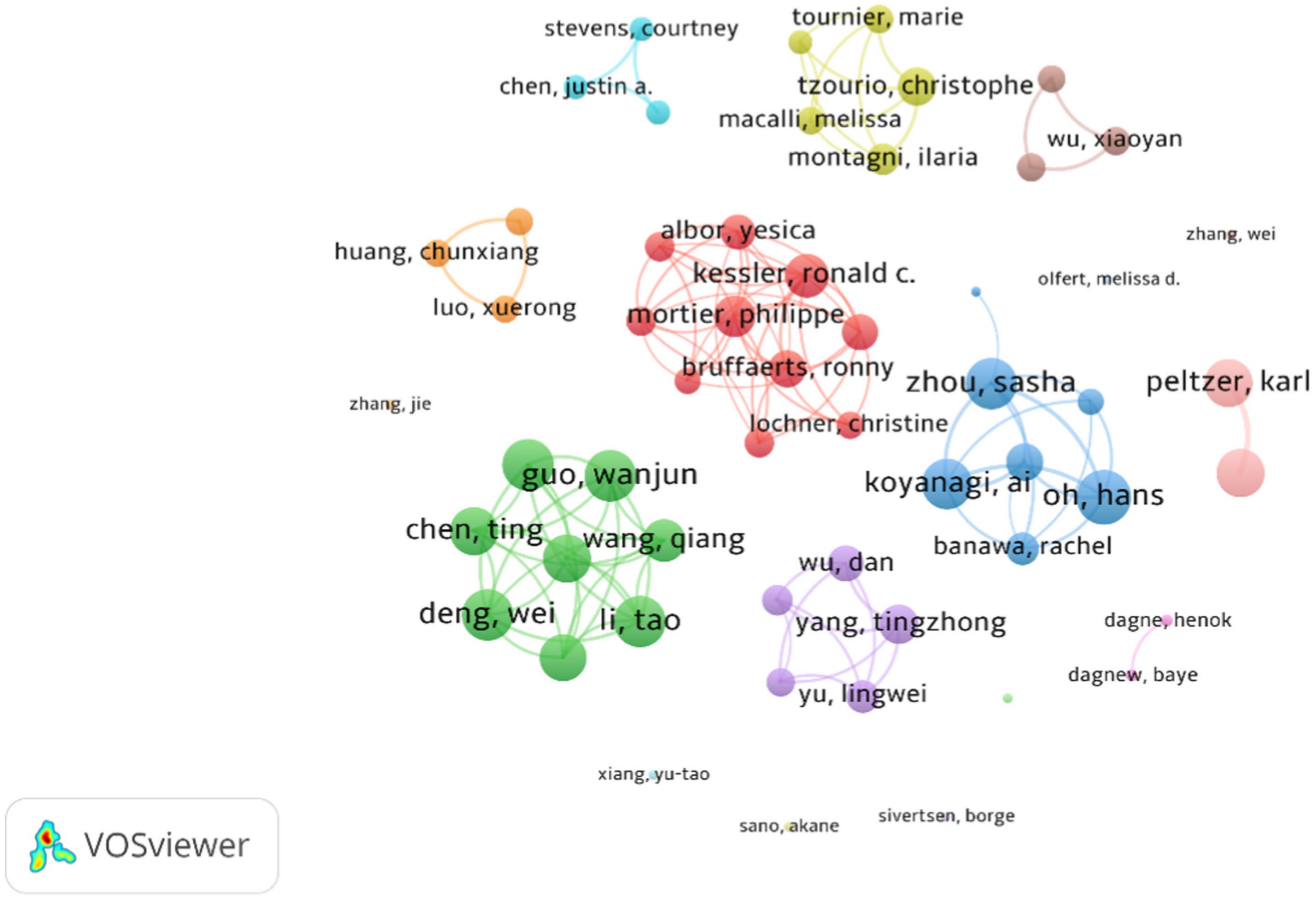
Visualization knowledge maps of the co-authorship between authors.

Figure 4 depicts the collaboration network among researchers, where nodes represent researchers, the node size indicates the quantity of publications by researchers, and lines between nodes denote collaboration relationships. This analysis includes only authors with a minimum of 5 articles in the dataset, resulting in 55 nodes and 113 connections. The graph reveals the presence of multiple collaborative teams, with the Red cluster led by Mortier, Philippe and the Green cluster led by Wang, Qiang emerging as the two largest collaborative teams in this field. Researchers within these teams exhibit close connections, indicative of robust collaboration. Additionally, there are other collaborative teams with five or more members, including the Blue cluster led by Oh, Hans, the Purple cluster led by Yang, Tingzhong, and the Yellow cluster led by Tzourio, Christophe. Meanwhile, independent authors appear less frequently in the graph, and the thickness of the lines signifies the degree of association between researchers, contingent on the frequency of their co-appearance in the same articles. The position of researchers in the clusters reflects their frequent and interconnected collaboration with other researchers, aligning with collaboration patterns observed in other bibliometric studies where researchers with social importance and productivity tend to enhance the productivity of their co-authors (Ho et al., 2017).

### 3.5 Analysis of co-citation sources network for AI in mental health of college students

**Figure 5:**
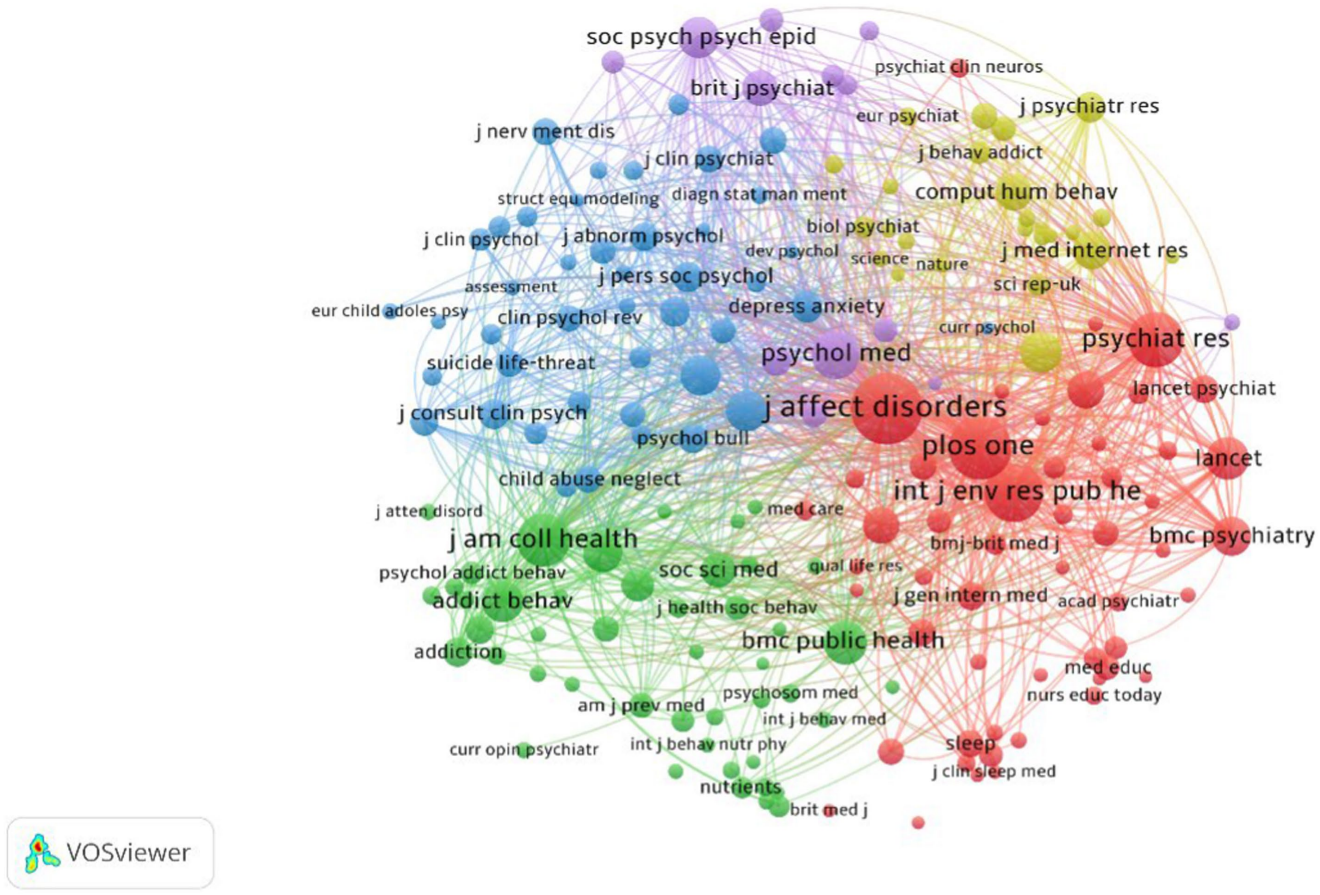
Visualization knowledge maps of the co-citation in journals.

The co-citation relationship between journals refers to two journals cited by the same third journal. The more frequently two journals are cited by other journals, the closer their co-citation relationship, and journals that are frequently co-cited are considered to have a strong theoretical and semantic foundation (Hernandez-Torrano et al., 2020). To comprehensively explore the disciplinary foundation of AI in the field of college student mental health, we conducted a co-citation analysis of journals in the dataset using VOSViewer. In this analysis, only journals cited at least 60 times were considered, resulting in 180 nodes and 15,268 connections, as show in Figure 5. The findings reveal that research on AI in the field of college student mental health encompasses multiple disciplines to some extent. Specifically, the knowledge in this field is constructed by integrating five interrelated disciplines. In the lower-right portion of the graph, the Red cluster predominantly brings together journals from the field of psychiatry, such as *Lancet, Psychiatry Research, BMC Psychiatry*, and others. In the lower-left portion, the Green cluster is mainly composed of journals from the fields of public health and behavioral science, including *BMC Public Health, Journal of American College Health, Addictive Behaviors,* and more. In the upper-right portion, the Yellow cluster signifies the contribution of computer science and interdisciplinary journals to this field, such as *Computers in Human Behavior, Journal of Medical Internet Research, Nature, Science,* and others. In the upper-middle portion, the Purple cluster integrates relevant journals from the field of psychology, such as *Psychological Medicine*. In the upper-left portion, the Blue cluster encompasses clinical psychology, child and adolescent psychiatry, and other psychiatry-related journals, including *Journal of Clinical Psychology, Clinical Psychology Review, European Child & Adolescent Psychiatry*, and more. In summary, research on AI in the field of college student mental health is interdisciplinary, primarily involving the integration of psychology, computer science, and public health.

### 3.6 Analysis of research hotspots and trend for AI in mental health of college students

**Figure 6:**
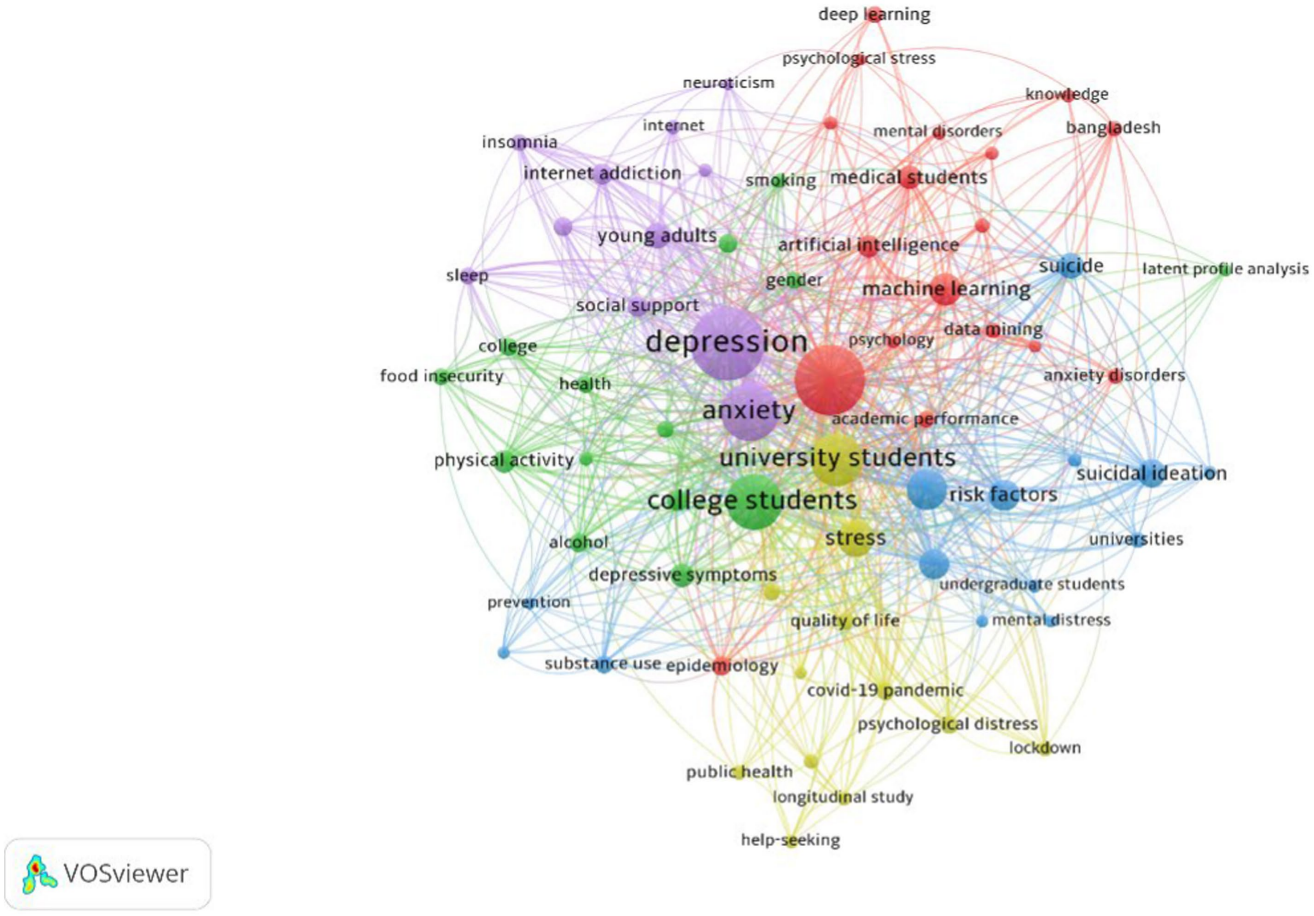
Visualization knowledge maps of keyword co-occurrence analysis.

To uncover the research hotspots in the field, we employed VOSviewer software for keyword co-occurrence analysis. Keyword co-occurrence refer to the frequency of two keywords appearing in the same article, and a network graph of keyword co-occurrence can delineate the research hotspots in a specific field. In this analysis, only keywords that appeared at least 10 times were considered, resulting in 82 nodes and 949 connections. Figure 6 illustrates the keyword co-occurrence graph and identifies five major research directions in the field of AI in college student mental health from 2003 to 2023.

The Purple cluster encompasses keywords like depression, anxiety, internet addiction, sleep, etc., representing the widespread attention of researchers to the impact of internet addiction on young people’s depression and anxiety. The Green cluster includes college students, depressive symptoms, physical activity, alcohol, etc., indicating that the mental health of college students is the second research hotspot in this field. The Yellow cluster comprises university students, stress, quality of life, COVID-19 pandemic, etc., suggesting that coping with the stress and quality of life of university students is the third research hotspot. The Blue cluster involves suicidal ideation, risk factors, mental distress, etc., representing factors posing a risk to the mental health of college students. The Red cluster involves keywords like mental health, machine learning, artificial intelligence, deep learning, etc., indicating that the application of AI technology in mental health is another widely discussed topic. Additionally, the five clusters are interconnected and closely related, signifying a close correlation among the five research directions.

**Figure 7:**
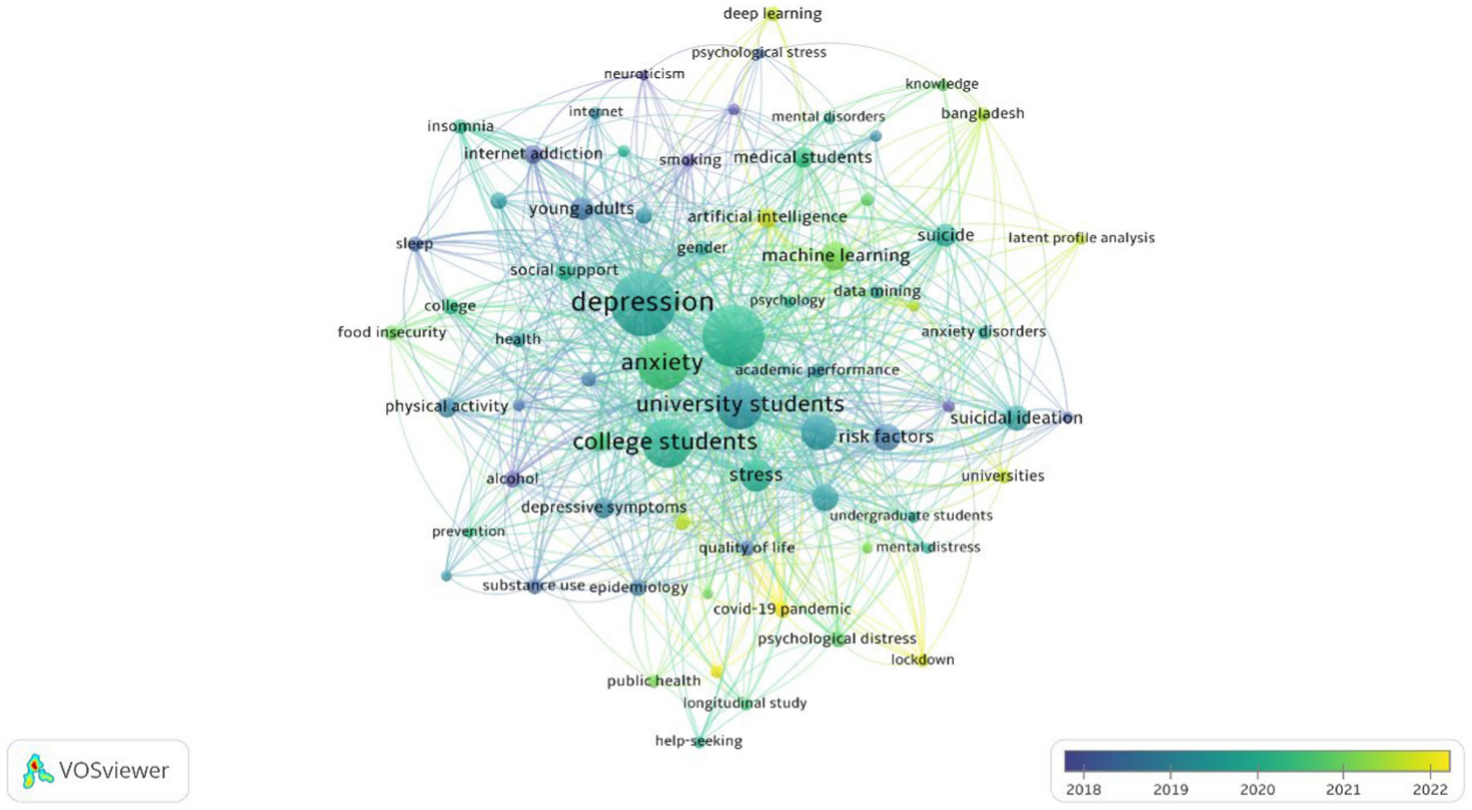
Visualization of keywords average year map from 2002 to 2022.

To explore the temporal trends of research hotspots in this field from 2003 to 2023, we conducted a time-based visual analysis of keywords, as depicted in Figure 7. Deep blue represents early research hotspots, yellow represents recent research hotspots, and the node size signifies the level of research intensity, with the legend indicating the average year. It is evident that early research hotspots in this field primarily focused on aspects such as sleep, internet addiction, alcohol, risk factors, etc. Around 2020, the research hotspots shifted toward college students, depression, anxiety, and related topics. By around 2022, keywords like “machine learning,” “artificial intelligence,” and “deep learning” represented recent research hotspots.

## 4 Discussion

### 4.1 General status and trends in AI in mental health of college students

This study employs bibliometric methods to explore the current status and trends of research on mental health and AI amomg college student by analyzing the development trends and distribution of publications in the WOS database, characteristics of research fields, scientific collaboration patterns, potential disciplinary compositions, and research hotspots and trends. In general, over the past two decades, the number of publications related to AI and college student mental health has steadily increased, reaching its peak in 2022, indicating a high research interest in this field. The journal with the most publications is the *Journal of Affective Disorders*, and *Psychiatry Research* has the highest average citation frequency. Psychiatry is the most significant research field. The IF and JCR partition are crucial indicators for assessing the quality of journals and the influence of papers. Papers published in high-IF journals or within the Q1/Q2 category are generally regarded as having higher academic standards and impact (Bornmann and Marx, 2016). In this study, a total of 721 journals published 1722 articles. The top 10 ranked journals published 452 articles, accounting for over a quarter of the total. Additionally, nine of these journals were positioned in either the Q1/Q2, and six had an IF exceeding 4. These data indicate that the data sources used are reliable and have a high academic level and attention. Additionally, China and the United States have made substantial contributions to the growth of publications in this field. Harvard University in the United States is the most influential institution, and the most prolific and impactful authors are Prof. Peltzer Karl and Kessler Ronald C. Keyword analysis indicates that the primary mental health issues faced by college students are depression, and the most widely used AI technology in college student mental health is machine learning, with the most applications being risk factors. Keyword co-occurrence analysis reveals that the research hotspots in this field mainly focus on the factors influencing college students’ mental health and the application of AI in college student mental health. Recently, high-frequency keywords such as “machine learning,” “artificial intelligence,” and “deep learning” represent the latest research trends and frontiers in this field.

Furthermore, collaboration in this field needs to be strengthened, including international and interdisciplinary collaboration. Figure 3 indicates that international collaboration in this domain is substantially shaped by language, geography, and cultural backgrounds. For example, the largest international collaboration network (Red cluster) comprises primarily 15 countries/territories from South America and Europe with similar cultural backgrounds, where English serves as the primary language. The second-largest international collaboration network (Green cluster) is comprised of 6 countries/territories from Asia, 3 from Europe, and 2 from Africa. The third-largest international collaboration network (Dark Blue cluster) encompasses 10 countries/territories from Asia, demonstrating geographic proximity. Collaboration mainly concentrates in Europe, the Americas, and Asia, with limited international collaboration among authors from developing countries, and even less contribution from authors in underdeveloped territories. Cross-national collaborative research has become a means to enhance research quality, increase resource utilization, and influence (Hsiehchen et al., 2015). The research gap between developing and developed countries and its impact on global health research trends cannot be ignored (Akinremi, 2011). Journal co-citation analysis shows that research in this field is, to some extent, interdisciplinary. Journals from various fields jointly constitute the disciplinary framework of AI and college student mental health research, primarily composed of psychology and computer science, supplemented by public health, behavioral science, and other health-related disciplines. Robust interdisciplinary partnerships integrating multilevel behavioral socioecological models with the molecular, cellular, and ultimately physiological bases of health and disease will contribute to breakthroughs in public health (Mabry et al., 2008). Therefore, we hope that in the future, developed countries can collaborate more with countries in underdeveloped territories in this field, and researchers from different disciplines can establish good collaborative relationships to produce higher-quality research results, making research in this field more universally applicable.

### 4.2 Risk factors affecting the mental health of college students

The co-occurrence analysis of keywords (Figure 6) reveals that the factors influencing college students’ mental health are one of the research hotspots in this field. Many factors contribute to the occurrence of mental health disorders in college students, including gender, genetics, personal factors, as well as family, school, and societal aspects (Beiter et al., 2015; Thompson et al., 2020; Seehuus et al., 2021). However, this study identified several interesting points.

Firstly, the keyword “internet addiction” in the Purple cluster caught our attention. Internet addiction profoundly affects the mental health of college students, as prolonged immersion in the virtual world can lead to anxiety, depression, and other psychological issues (Shen et al., 2020). A meta-analysis involving 33,650 college students showed a positive correlation between mobile phone addiction (MPA) and anxiety, depression, and sleep disturbances (Li et al., 2020), suggesting that students with MPA are more prone to high levels of anxiety and depression. Logistic analysis by Lin et al. (2019) confirmed a significant negative correlation between the degree of internet addiction and sleep quality, indicating that poor sleep quality increases the risk of psychological disorders in college students (Nyer et al., 2013). Additionally, the impact of internet addiction on college students’ mental health is evident in social isolation and self-identity. The popularity of social media applications has reduced face-to-face communication time in the real world, leading to social isolation in males who engage in online gaming and affecting self-identity in females who use the internet to avoid exclusion from online interactions. The social standards and values in the virtual world can influence college students’ self-identity, causing confusion and negative emotions (Tateno et al., 2019; Kato et al., 2020; Herruzo et al., 2023). Furthermore, research indicates a moderate negative correlation between exercise and internet addiction in young people, with the relationship’s strength unaffected by data collection time, country or region, or population type (Xiao et al., 2022). Exercise therapy has been shown to be effective in treating internet addiction in college students (Zhang et al., 2023). Therefore, incorporating regular physical activity into higher education policies or encouraging students to engage in sports can reduce the potential threat of internet addiction to mental health.

Secondly, the keyword “alcohol” in the Green cluster is another factor influencing the mental health of college students. Excessive alcohol consumption may lead to psychological problems such as anxiety and depression in college students (Schry and White, 2013). The impact of alcohol on college students’ mental health may primarily stem from its damage to the brain and nervous system. Long-term alcohol abuse can affect normal brain function, leading to cognitive decline, impacting academic performance, and increasing psychological burden (Meda et al., 2017; Zahr and Pfefferbaum, 2017). Additionally, researchers have identified the liver aldehyde dehydrogenase 2 (ALDH2) in the brain’s astrocytes as a primary mechanism for the direct regulation of alcohol-induced behavioral functional impairment. This provides strong evidence for the role of the brain in alcohol metabolism (Jin et al., 2021). Leclercq et al. (2020) transplanted the intestinal microbiota of alcohol use disorder patients into mice, resulting in significant social disorders in mice, demonstrating the association between gut microbiota imbalance and changes in brain alcohol metabolism. These changes, in turn, were related to a decrease in the synthesis of β-hydroxybutyrate (BHB) in the liver, hindering the neuroprotective effects of BHB. Moreover, alcohol abuse may disrupt sleep, affecting emotions and cognitive abilities, with negative effects on mental health (Lund et al., 2010). In summary, the harm caused by alcohol to college students’ mental health should not be overlooked. To maintain mental health, students should be vigilant about alcohol consumption, adopt a healthy lifestyle, seek social support, and, when necessary, seek professional psychological counseling. Through positive attitudes and scientific psychological adjustment methods, college students can better cope with the psychological challenges alcohol may pose.

Thirdly, the impact of the keyword “covid-19 pandemic” in the Yellow cluster on the mental health of college students is equally noteworthy. The COVID-19 pandemic and associated lockdowns have profoundly affected the mental health of college students, with the uncertainty and fear brought about by the pandemic leading to widespread anxiety and stress among students (Singh et al., 2020). A survey targeting college students revealed that, out of 7,143 respondents, 0.9% experienced severe anxiety, 2.7% experienced moderate anxiety, and 21.3% experienced mild anxiety (Cao et al., 2020). A broader survey reported that approximately one-fifth of Chinese college students exhibited symptoms of anxiety during the COVID-19 pandemic (Fu et al., 2021). Economic impacts, disruptions to daily life, and delays in academic activities emerged as major sources of psychological stress, posing a severe threat to the mental health of college students (Cao et al., 2020). Moreover, the COVID-19 pandemic resulted in social isolation, depriving college students of normal social activities. Online learning and restricted living conditions led to feelings of loneliness and social isolation among students, preventing them from enjoying traditional campus life. Prolonged social deprivation may contribute to the accumulation of emotional issues, negatively impacting the mental health of college students (Lim et al., 2022; Sun et al., 2023). Additionally, the pandemic heightened stress and burdens for college students, who may experience increased academic pressure and uncertainties about the future, leading to psychological issues such as anxiety and depression (Robledo-Martin et al., 2023) To safeguard the mental health of college students, it is crucial to adopt proactive psychological adjustment strategies. Recommendations include maintaining social connections, seeking social support, adopting a healthy lifestyle, and learning effective stress management and emotional regulation methods. The availability of mental health services in higher education institutions is also an essential resource, providing professional assistance and support to help students navigate through this challenging period (Oswalt et al., 2020; Kihumuro et al., 2022).

### 4.3 Application of AI in mental health of college students

The co-occurrence analysis of keywords (Figure 6) reveals that the Red cluster signifies the application of AI technology in the field of mental health, representing another research hotspot. The keyword visualization analysis over time (Figure 7) identifies “machine learning,” “artificial intelligence,” and “deep learning” as the latest research trends and frontiers in this field. Through comprehensive data analysis and consensus among two article reviewers, we have identified four directions for AI in the domain of college students’ mental health: (1) prediction and diagnosis; (2) treatment; (3) prognosis; (4) ethical considerations. The following sections will discuss each category in detail.

#### 4.3.1 Prediction and diagnosis

AI has shown great promise in predicting and diagnosing critical events in mental health. The feasibility of AI prediction regarding critical events associated with various healthcare issues, such as hypertension, diabetes, and heart failure, has been demonstrated (Ye et al., 2018; Arcadu et al., 2019; Krittanawong et al., 2020). In the field of mental health, AI technology can establish predictive models by analyzing big data to assess the mental health status of participants, thereby effectively saving the currently limited resources in psychological education and medical development, making psychology a more predictive science (Yarkoni and Westfall, 2017). For instance, Garriga et al. (2022) developed an electronic health record-based continuous risk prediction for various mental health crises using the XGBoost algorithm. The sensitivity and specificity for crisis prediction were 58 and 85%, respectively, indicating potential clinical value. Natural Language Processing (NLP) techniques identified symptoms of severe mental illnesses from electronic health record data (precision = 90%; recall = 85%) (Jackson et al., 2017). Kalmady et al. (2019) proposed the integrated model EMPaSchiz, achieving an accuracy of 63–71% in identifying neuroanatomical subtypes of schizophrenia, surpassing early machine learning models for diagnosing schizophrenia. Additionally, Mahalingam et al. (2023) employed logistic regression, multilayer perceptron (MLP) neural network, support vector machine (SVM), random forest (RF), and XGBoost algorithms to predict anxiety in 329 college students, with the MLP model achieving the highest accuracy (AUC = 80.70%), highlighting self-rated health as the primary predictor of anxiety. In summary, these studies exemplify the excellent potential of AI in the field of mental health prediction. However, to avoid data overfitting and over interpretation of preliminary results, more work is needed to bridge the gap between AI research and clinical care in college students’ mental health.

Currently, traditional diagnostic methods face challenges in diagnosing mental health issues among college students due to individual differences, diverse expressions, and the concealment of psychological problems. Moreover, limited mental health resources and a shortage of professionals hinder timely diagnosis and intervention for college students’ mental health issues (Woodhead et al., 2021). AI technology offers new possibilities to address these challenges. Magnetic Resonance Imaging (MRI) as an objective biological marker, combined with AI technology, has made breakthroughs in researching mental illnesses like depression (Kragel et al., 2018). Deep learning technology, by learning inherent patterns and hierarchical representations from sample data, enables machines to recognize data such as text, images, and sounds, and MRI based on deep learning technology has proven effective in diagnosing and analyzing severe depression (Wang et al., 2021). Considering that bipolar affective disorder is often misdiagnosed as severe depression, researchers used the SVM algorithm to learn brain signals, achieving a combined accuracy of 75% in identifying bipolar affective disorder and severe depression (Rubin-Falcone et al., 2018). This suggests that integrating AI into neuroimaging technology holds promise for improving the identification of objective biological markers and the diagnosis of mental illnesses. Furthermore, researchers developed a stacked support vector machine model based on behavioral data from 137 college students, achieving an accuracy of 89.4% in the early diagnosis of Persistent Depressive Disorder (PDD) (Upadhyay et al., 2023). Katsis et al. (2011) identified individuals’ emotional states based on five pre-defined categories (relaxation, neutral, surprise, anxiety, and extreme anxiety) by collecting physiological data (blood volume pulse, heart rate, skin conductance response, and respiration) of anxiety patients. Using four different classification algorithms (artificial neural network, support vector machine, random forest, and neural fuzzy system), the overall classification accuracy was 84.3%. These studies suggest that machine learning techniques applied to behavioral and physiological features can be used for the early identification of abnormal emotions, aiding in the timely detection of potential psychological disorders among college students and assisting in clinical decision-making in cases of uncertain diagnosis.

#### 4.3.2 Treatment

Currently, interventions for college students’ mental health disorders mainly include pharmacotherapy and clinical psychotherapy; however, the effectiveness of these treatment modalities is limited and may even lead to intolerable side effects (Iversen et al., 2018). Therefore, AI-based psychotherapy and interventions have begun to address these issues.

Firstly, applying AI technology to intelligent analysis of data through wearable devices is conducive to the automation and integration of real-time monitoring, diagnosis, and intervention of college students’ mental health. For example, Millings et al. (2015) used wearable sensors to intervene in students’ stress levels, and based on detailed reports from sensors and biofeedback training, found a reduction in stress and decreased depressive symptoms after the intervention. Chen et al. (2022) designed a rapid assessment and early warning system for depression in college students based on sensors and daily health data using Logistic and multilayer perceptron models. This system provides timely psychotherapy and intervention for students in the early stages of depression, preventing tragedies caused by mental health problems. With the improvement of prediction accuracy and the popularity of mobile devices, researchers have transformed models into complete early warning and intervention systems, embedded in mobile and wearable devices, deeply integrated into individuals’ daily lives, achieving real-time personalized interventions.

Secondly, in clinical practice, AI technology can predict and guide the efficacy of depression treatment plans based on patients’ baseline characteristics before treatment. Chekroud et al. (2016) used the gradient boosting machine algorithm to predict the response of patients with major depression to drug treatment. The predictive model they established had a classification accuracy of 60.0% for predicting the response of patients with major depression to escitalopram, with slightly lower predictive effects for other drugs. Although the overall accuracy is not high, this study reveals the potential of using machine learning to prospectively predict patients’ clinical efficacy. Benjet et al. (2023) developed a personalized treatment rule based on rich baseline predictive factors, using machine learning methods for the selection between guided internet-delivered cognitive-behavioral therapy (i-CBT) and self-guided i-CBT. The study, including students seeking anxiety or depression treatment in Colombia and Mexico, found that for most participants, guided i-CBT had a higher probability of anxiety and depression relief, while for some participants, self-guided i-CBT performed better in depression relief. Although these differences were not significant in terms of anxiety, they provide information for optimizing the allocation of guided i-CBT and self-guided i-CBT in resource-constrained environments.

Finally, in addition to treatment detection, advances in AI in the field of cognitive neuroscience also provide a feasible approach for mental health disorders. Multivoxel Pattern Analysis (MVPA) is considered a promising technique that introduces machine learning into the field of psychology, currently used to study information contained in distributed neural activity patterns to infer the functional roles of brain territories and networks (Mahmoudi et al., 2012). In application, based on MVPA, neural feedback decoding (DecNef) can be performed for identified brain activity patterns, guiding clinical interventions for mental illnesses (Cortese et al., 2021). Currently, neuroregulation techniques based on brain activity pattern decoding mainly include invasive and non-invasive techniques (Temel et al., 2012). Electroconvulsive therapy (ECT), as a representative of non-invasive neuroregulation techniques, remains one of the fastest and most effective methods in the treatment of depression, with a treatment response rate of 70% ~ 80% (Read and Bentall, 2010). Kaster et al. (2021) used logistic regression analysis to identify patient-level characteristics of hospitalized depression patients receiving ECT across various age groups using the generalized estimating equation method. They found associations between age, marital status, education level, and ECT utilization. With the development of information technology, modern neuroregulation methods have become more intelligent. The Accelerated Intelligent Neuromodulation Therapy (SAINT) developed by Stanford University utilizes an accelerated high-dose brain stimulation (iTBS) protocol combined with functional magnetic resonance imaging (fcMRI) for personalized targeting, effectively relieving depressive symptoms (Cole et al., 2020). A clinical double-blind randomized controlled trial showed that after 4 weeks of SAINT treatment, participants’ depression symptom scores decreased by an average of 52.5% from baseline, while the control group only showed a 11.1% reduction (Cole et al., 2022). Additionally, SAINT was also effective in the treatment of obsessive-compulsive disorder (Williams et al., 2021). SAINT provides an effective, rapid, safe, and tolerable novel treatment approach for college students’ mental health issues. However, there are currently no cases of SAINT applied to college student samples, and more research is needed to confirm its long-term efficacy and safety.

#### 4.3.3 Prognosis

In the research and clinical practice of mental disorders, determining individual prognosis is crucial for management, psychological education, and providing preventive treatment and interventions. The lack of accurate early classification may lead individuals who do not require treatment to undergo unnecessary interventions, resulting in a waste of medical resources (Siskind et al., 2016). Therefore, effective prognosis prediction will contribute to improving later treatment plans. In terms of predicting disease progression, the application of AI technology has shown significant prospects. Basaia et al. (2019) used convolutional neural networks to predict the conversion from mild cognitive impairment to Alzheimer’s disease, achieving a prediction accuracy of over 75%. Yuan et al. (2018), based on resting-state functional MRI features from 22 patients with post-traumatic stress disorder (PTSD) treated with paroxetine, established a model using the support vector machine method to predict the long-term clinical outcomes of PTSD patients with an accuracy of 72.5%. This is crucial for defining potential biomarkers to tailor PTSD treatment and improve prognosis.

In the treatment of depression, researchers employed a machine learning approach, combining data fusion from structural and functional task-based MRI, to establish a prediction model that can predict the trajectory of depression over 2 years (chronic, gradually improving, and rapid remission) based on neural responses to emotional facial expressions. The predictive rates are similar to the aforementioned examples (Schmaal et al., 2015). Additionally, using a machine learning approach with generated embeddings (GE), Fraessle et al. (2020) built a model based on MRI data from 85 patients with major depressive disorder (MDD), achieving a prediction accuracy of 79% for chronic depression, while the average accuracy for predicting progressive improvement and rapid remission was only 61%. Although the results are not entirely convincing, they provide preliminary evidence for the potential of GE in obtaining clinically interpretable predictions in terms of network mechanisms. In summary, these studies highlight the ability of AI and machine learning technologies to optimize prognosis assessment through individual stratification. This will contribute to improving the accuracy of prognosis prediction for mental health disorders in college students, promoting personalized treatment outcomes, and enhancing a more comprehensive understanding and management of mental health issues.

#### 4.3.4 Ethical considerations

As mentioned earlier, AI technology has demonstrated significant advantages in the field of college student mental health. It has achieved commendable results in clinical practice and medical research by analyzing extensive clinical diagnostic and prognostic data, with groundbreaking implications deserving greater attention. However, researchers or healthcare professionals involved in the development, selection, testing, and implementation assessment of AI technology must be aware of ethical challenges, including biases in clinical data and the associations between mental illnesses and factors such as race and gender (Chen et al., 2019). Recognized ethical principles guiding biomedical research, including autonomy, beneficence, and justice, must be prioritized and, in certain cases, strengthened (Raymond, 2019). Furthermore, the use of AI in the field of mental health may raise concerns about students’ personal privacy, as the collection and analysis of individual mental health data may lead to information leakage and trigger worries and anxieties (Lee et al., 2021). Lucas et al. (2014) pointed out that patients in mental health clinical practice often hesitate to provide sincere responses. Participants interacting with virtual humans (VHs) experience lower fears of self-disclosure, aiding in overcoming significant barriers to obtaining authentic patient information. The introduction of AI as a new clinical tool in medical practice has brought about profound changes in doctor-patient relationships and promoted the idea of establishing a new clinical model involving third-party participation. This relationship is crucial for clinical practices in psychiatry, and discussions should be held regarding the use of data processing and the protection of patient privacy.

Additionally, apart from considering issues related to patients’ personal privacy, the application of AI in college student mental health should also incorporate specific preventive measures to avoid excessive reliance and clinical misuse. Researchers utilized automated voice analysis combined with machine learning to predict the likelihood of later development of psychosis in 34 adolescents at clinical high risk (CHR), achieving 100% accuracy in predicting later psychosis development (Bedi et al., 2015). However, due to the small sample size in this study, there may be a risk of model overfitting, necessitating cautious interpretation of the results and further research, while also considering potential ethical concerns. Martinez-Martin et al. (2018) questioned the ethical appropriateness of using machine learning for prognosis estimation in treating mental illnesses, as it is unknown whether variables affecting model effectiveness, such as differences in mental practices and social support, exist in local environments. Addressing these issues, Char et al. (2020) proposed a conceptual model for machine learning healthcare applications (ML-HCA) from conception, development, to implementation, providing a systematic ethical assessment approach to understand and manage ethical issues associated with ML-HCA and advocating for interdisciplinary collaboration among stakeholders. Finally, the application of AI in the field of mental health should not lead to the deprivation or replacement of the authority of mental health professionals or hinder communication between clinical doctors and patients. Instead, AI should be considered a tool that supports clinical practices and medical research in college student mental health.

### 4.4 Strengths and limitations in our analysis process

#### 4.4.1 Strengths

The Web of Science database is a globally authoritative citation database that includes a series of core journals with the most academic impact in various disciplines. These documents, to some extent, can reflect the global trends in the forefront of specific disciplines or fields. By examining the quantity and quality of scientific materials produced, we can objectively assess the current status and level of scientific progress (Tran et al., 2019). This study is based on the WoS core database, representing the first bibliometric analysis of literature in the field of AI and college student mental health. Through visual analysis, we present the development trends in this field, the distribution of publications, reveal scientific collaboration networks among authors and countries, clarify the disciplinary composition of the field, and analyze and discuss the research on AI and college student mental health. The future of AI in college student mental health is promising and holds significant research potential. Therefore, this study provides insights for future directions and research methods in this field.

#### 4.4.2 Limitations

However, this study has some limitations. Firstly, the use of the Web of Science (WoS) core database as the source of literature may introduce significant differences since various databases exhibit variations. If the literature is obtained from another database, more changes in citations or articles may be observed (Bayram et al., 2016), limiting the generalizability of the study results. Most importantly, some key terms, although listed as popular keywords, lack informative content themselves (e.g., “health,” “psychology”), making analysis impossible. Thirdly, we only collected English literature retrieved from the WoS core database; therefore, we did not include all literature in this field, potentially affecting the summarization and generalization of the results. Fourth, research areas may have nuanced differences and interpretations. Some studies may cross disciplines or not fit predefined categories, highlighting the limitations of methods based solely on journal names or stated aims to define each paper’s research domain (Zhang et al., 2022). Despite these limitations, our analysis offers valuable insights into trends and prevalent research areas at the intersection of AI and college student mental health.

Despite the potential of AI in the research and treatment of college student mental health, there remain many challenges and difficulties to address. For instance, collecting a sufficient amount of high-quality data for training and validating machine learning algorithms may be challenging. Furthermore, the use of AI in college student mental health may pose risks like privacy breaches, algorithmic bias, and misuse of AI tools. This could weaken interpersonal communication and professional support, impacting personalized care. Thus, implementing regulations and safeguards is crucial to maximize benefits for students while minimizing risks. Nevertheless, we believe that the bibliometric methods employed in this study offer new insights into the development and current status of the field, highlighting some challenges hindering its progress, and can provide inspiration for further research in this area.

## 5 Conclusion

In summary, the results of this study reveal the trends in the development of the field over the past 20 years, offering insights into understanding the scientific patterns between AI and college student mental health on a global scale. Firstly, over the last two decades, researchers have shown increased interest in the study of AI and college student mental health. The sustained research momentum in recent years suggests that this field is still evolving and is likely to continue developing in the future. Secondly, given the limited transnational collaborative research in this field and its interdisciplinary nature, it is essential to break down barriers between countries, encourage collaboration among researchers and nations, and call for engagement from researchers in psychology, computer science, public health, and other related fields to participate in the study of AI and college student mental health. This will promote global research in this field and collectively contribute to the maintenance of college student mental health. Thirdly, researchers have conducted in-depth investigations into various aspects related to AI and college student mental health over the past 20 years, achieving notable progress, particularly in identifying risk factors affecting college students and the application of AI in college student mental health. Fourthly, the causes of mental health issues are diverse, and traditional psychological research often stems from top-down theory-driven approaches that require well-formulated theoretical hypotheses validated through data collection. In contrast to traditional psychological research methods, future AI-driven research can adopt a bottom-up, data-driven approach to uncover relationships between variables, revealing previously overlooked causes of college student mental health issues. This approach is more conducive to developing intelligent applications for prevention and intervention with enhanced effectiveness. Fifthly, comprehensive integration and development of AI technology with psychological research involve merging psychology with knowledge from disciplines such as biology, society, information, and computer science. This includes the development of adaptive learning algorithms, close attention to machine predictions, and ethical supervision to leverage big data in mental health for the benefit of a broader population. Additionally, this paper also serves as a valuable resource for psychological experts without an AI background or individuals interested in the application of AI in the field of college student mental health but not familiar with it. In conclusion, this study indicates that AI plays an important role in promoting the mental health of college students, including using AI technology to identify and predict potential mental health risk factors, assisting psychological experts in rapid and accurate diagnosis, providing personalized treatment plans for students, and supporting research on mental health. It will aid researchers and healthcare institutions in better understanding the development of AI in college student mental health research and its potential practical significance.

## Data availability statement

The raw data supporting the conclusions of this article will be made available by the authors, without undue reservation.

## Author contributions

JCh: Conceptualization, Data curation, Formal analysis, Investigation, Methodology, Software, Supervision, Validation, Writing – original draft, Writing – review & editing. DY: Conceptualization, Data curation, Formal analysis, Investigation, Methodology, Software, Supervision, Validation, Writing – original draft, Writing – review & editing. RD: Formal analysis, Methodology, Software, Writing – review & editing. JCa: Formal analysis, Methodology, Software, Writing – review & editing. ZA: Conceptualization, Funding acquisition, Project administration, Writing – review & editing. SZ: Conceptualization, Funding acquisition, Project administration, Writing – review & editing.
